# Novel synchronous nasal involvement of inverted papilloma and recurrent respiratory papillomatosis with confirmed human papillomavirus isolated from nasal septum and middle turbinate: a case report

**DOI:** 10.1186/s13256-019-2153-1

**Published:** 2019-07-15

**Authors:** Jeremie D. Oliver, Neil S. Patel, Dale C. Ekbom, Janalee K. Stokken

**Affiliations:** 1 Mayo Clinic School of Medicine, Mayo Clinic, Rochester, MN USA; 2 Department of Otorhinolaryngology, Mayo Clinic, 200 1st St SW, Rochester, MN 55905 USA

**Keywords:** Inverted papilloma, Recurrent respiratory papillomatosis, Sinonasal tumors, Human papillomavirus, Head and neck surgery

## Abstract

**Background:**

Recurrent respiratory papillomatosis is a chronic disease of viral origin affecting the larynx, trachea, and lower airways. Inverted papilloma, most commonly originating from the lateral nasal wall, is typically a single, expansile, locally aggressive tumor that remodels bone around the site of origin.

**Case presentation:**

We report a case of histopathologically proven inverted papilloma occurring in a 50-year-old Caucasian man with recurrent respiratory papillomatosis affecting his nasal cavity, larynx, and trachea. This constitutes the first report of nasal involvement in recurrent respiratory papillomatosis. Viral *in situ* hybridization studies demonstrated evidence of human papillomavirus in both the septum and middle turbinate subsites. Repeat nasal excision with margin analysis is planned.

**Conclusions:**

This report emphasizes the importance of considering a broad differential diagnosis in patients with papillomata, and obtaining comprehensive histopathologic evaluation of lesions in multiple subsites in order to rule out inverted papilloma or overt malignant transformation, particularly if high-risk human papillomavirus (HPV) subtypes are identified.

**Level of evidence:**

4

## Introduction

Recurrent respiratory papillomatosis (RRP) is a chronic disease of viral origin most commonly affecting squamous and ciliated epithelia of the larynx, trachea, and lower airways, particularly at the laryngeal ventricle and carina [1]. The bimodal age distribution reflects two discrete disease subtypes: juvenile-onset RRP and adult-onset RRP. In the former, vertical transmission of human papillomavirus (HPV) from mother to child results in predominantly voice and airway manifestations that present around the age of 5. The etiology of adult-onset RRP is less clearly understood; however, it is also caused by low-risk HPV subtypes 6 and 11. For papillomatosis confined to the larynx, serial endoscopic debulking achieves disease control in the majority of patients. However, patients who develop tracheobronchial disease may need more frequent or adjuvant interventions, such as cidofovir or bevacizumab injection [2]. Nasal extension of RRP has not been reported in the literature. Common symptoms of RRP range from voice changes to life-threatening airway compromise, often requiring repeat endoscopic procedures to remove the obstructing lesions and preserve normal tissue and function [3].

We present this case of histopathologically proven inverted papilloma (IP) occurring in a patient with RRP affecting the nasal cavity, larynx, and trachea. This constitutes the first report of histopathologically confirmed nasal involvement in RRP.

## Case presentation

A 50-year-old Caucasian man with adult-onset RRP was referred for rhinology consultation for multiple unilateral nasal papillomata. He had undergone endoscopic potassium titanyl phosphate (KTP) laser ablations for diffuse laryngotracheal disease nearly every 3 months prior. Previous biopsies showed evidence of moderate to severe dysplasia on some of the tracheal lesions. Repeat airway debulking and nasal biopsy at our institution revealed squamous papillomata without evidence of dysplasia in both specimens. *In situ* hybridization (ISH) studies for HPV were negative for high-risk subtypes.

**Fig. 1:**
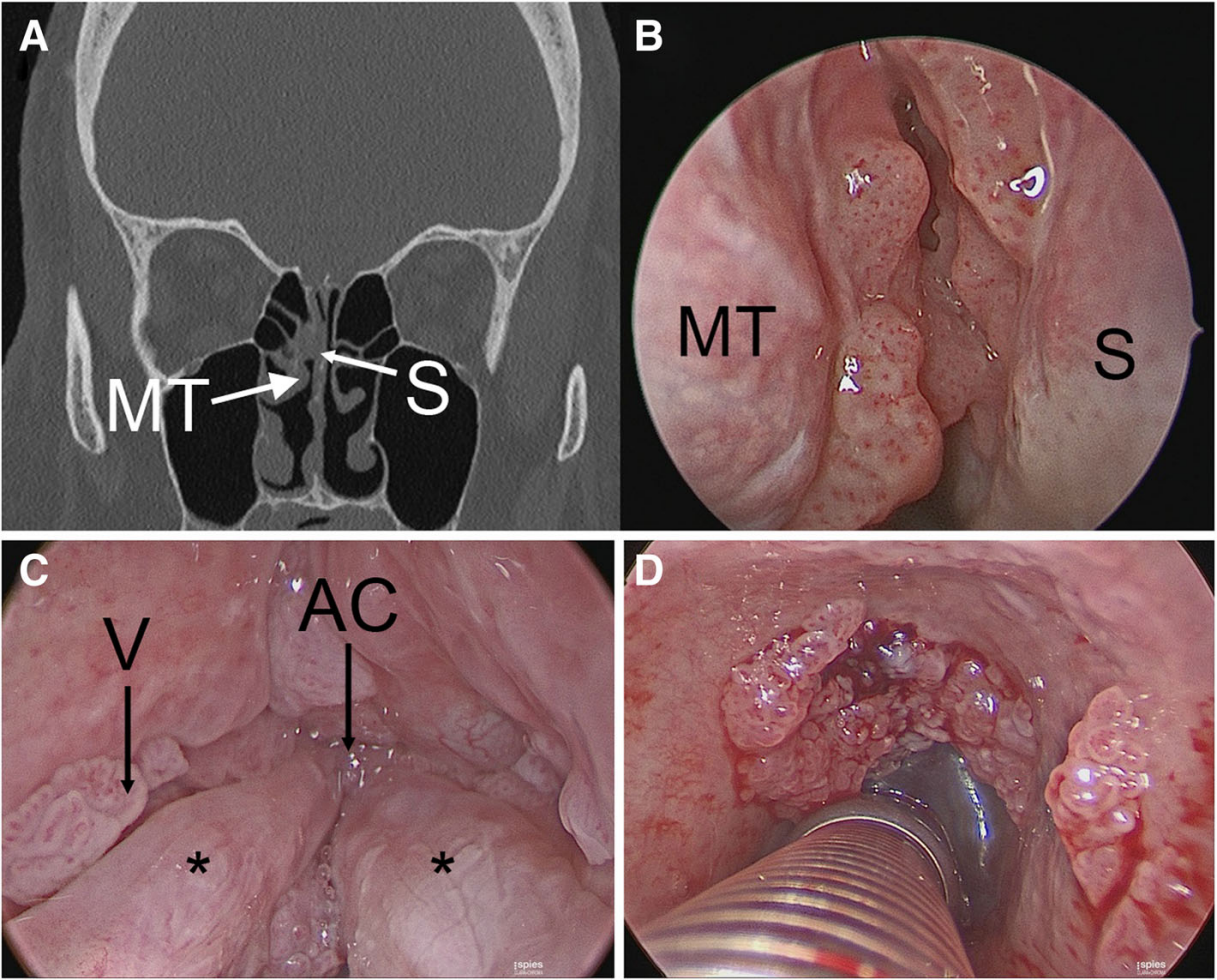
Non-contrast coronal computed tomography demonstrating soft tissue nodularity along medial aspect of middle turbinate and posterior septum (panel **a**) with corresponding endoscopic view (panel **b**). Diffuse laryngotracheal papillomatosis involving ventricle, anterior commissure, true vocal folds (panel **c**, *asterisks* denote true vocal folds), subglottis, and trachea (panel **d**). *AC* anterior commissure, *MT* middle turbinate, *S* septum, *V* ventricle

Three months later, his examination exhibited increased nasal involvement with lesions of the middle and superior turbinates and posterior septum while sparing his nasopharynx. Computed tomography (CT) imaging showed mucosal irregularities consistent with examination findings without hyperostosis or posterior ethmoid involvement (Fig. 1).

**Fig. 2:**
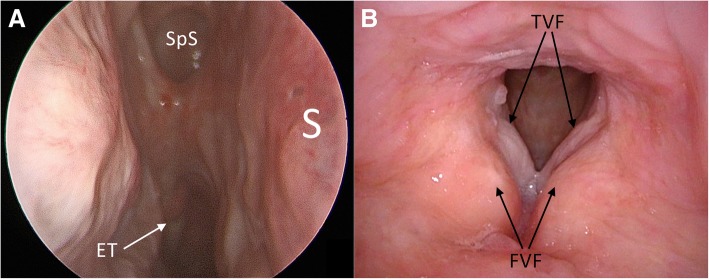
Endoscopic view of right nasal cavity (panel **a**) and larynx (panel **b**) 8 months after papilloma excision. *ET* eustachian tube, *FVF* false vocal fold, *S* nasal septum, *SpS* sphenoid sinus, *TVF* true vocal fold

Intraoperative frozen sections were consistent with benign respiratory papilloma. However, permanent section pathologic analysis revealed inverted type Schneiderian papilloma in all nasal specimens and benign squamous papillomata with mild to moderate atypia in the larynx and trachea. His surgical resection included removal of the majority of the lesion with an attachment site centered on the middle turbinate. The mucosal surface of the posterosuperior septum and a small portion of skull base were also removed, as they were involved with papilloma (see Fig. 2). It is unclear if this patient exhibited synchronous IP of the middle turbinate and recurrent respiratory papilloma of the septum and skull base, or if the multisite disease represents diffuse nasal IP. Viral ISH studies demonstrated evidence of HPV family 6 (subtypes 6 or 11) in both the septum and middle turbinate subsites. Repeat nasal excision with margin analysis is planned in conjunction with the next laryngeal procedure as frozen section pathology was equivocal and the lesion encroached on his skull base.

Prior to coming to our institution, he underwent previous treatments that included KTP laser, cidofovir injections, and indole-3-carbinol. He did endorse a history of smoking cigarettes and has recently decreased consumption from one pack per day to half a pack per day. Based on the initial presentation and pertinent medical history, our patient is at increased risk for malignant transformation of papillomata, particularly in the larynx [4]. Now at 12 months postoperatively, he is stable with no signs of residual malignancy.

## Discussion

The case presented in this report is the first of its kind to demonstrate IP occurring within the context of RRP. Furthermore, the same strain of HPV was isolated in both lesions, providing support to the theory of HPV involvement in the pathogenesis of both IP and RRP lesions.

While single, exophytic papillomata in the nasal vestibule are fairly common, diffuse intranasal papillomatosis has only been reported in cases without coexistent RRP. Recurrence was noted soon after initial resection in two cases, suggesting a natural history similar to RRP [5]. Although RRP is classically described as a benign neoplasm of the airway caused by low-risk HPV viral subtypes, transformation to dysplasia and invasive carcinoma can still occur [4]. The rate of dysplasia in patients with adult-onset RRP is roughly 10%, with malignancy developing in up to 5%; however, rates of dysplasia can reach as high as 55% in recent studies reported in the literature; some authors suggest that HPV-*negative* papilloma, tobacco use, and previous cidofovir administration may increase the risk of malignant transformation [4]. In contrast, laryngeal squamous cell carcinoma caused by HPV infection is typically associated with high-risk subtypes 16 and 31, among others [6].

Schneiderian papilloma refers to all squamous papillomata of the nasal or paranasal sinus mucosa. The inverted type, most commonly originating from the lateral nasal wall, are polypoid lesions where metaplastic squamous epithelium replaces and interdigitates with respiratory pseudostratified ciliated columnar epithelium, thus creating multiple squamocolumnar junctions; this mechanism of development has been correlated in previous studies with the similar metaplastic transformation zone that forms on the cervix with the progression of cervical cell turnover, and subsequent increased susceptibility to neoplastic cell transformation [7, 8]. The rate of transformation of IP into squamous cell carcinoma is probably between 5 and 15% [9, 10]. Malignancy in this setting can be categorized as synchronous carcinoma alongside IP, focal invasive carcinoma within IP, and complete transformation of a recurrent IP into squamous cell carcinoma [10]. Recent studies have reported a significant increase in both recurrence and malignant transformation in HPV-infected IPs; some data support an association between the presence of high-risk subtypes and malignant transformation [8].

While there continues to be contention in the existing literature as to the existence of malignant transformation of HPV-infected IPs and RRP lesions, this report provides histopathologic support for the hypothesis that such an association exists. Further studies are warranted to determine the potential role of HPV in the pathogenesis of both disease processes.

## Conclusions

This represents the first report of IP occurring within the context of RRP. Interestingly, the same strain of HPV was isolated in both the IP and RRP lesions. The proposed pathogenesis of HPV in IP lesions remains controversial in the literature, and is less known currently than the mechanism of HPV driving the transformation of IP to squamous cell carcinoma. Our case provides support to the theory of HPV involvement in the pathogenesis of both IP and RRP lesions. We were unable to find any additional reported cases of diffuse sinonasal papillomata.

Key lessons learned from this case include the importance of histopathologic analysis of papillomata to rule out the presence of alternative subtypes, dysplasia, or malignant transformation. Furthermore, the identification of this lesion was confounded by coexistent laryngotracheal disease, an uncommon attachment site, previous negative biopsies, and absence of classic imaging features. ISH studies to determine HPV subtype are valuable in assessing the risk of dysplastic conversion. Wide excision of sinonasal papillomata with tumor-free margins is recommended to decrease the risk of recurrence.

## Data Availability

Data and material used for this study were stored in a secured, internal database which was de-identified for the purpose of this study.
